# Prenatal and early life exposure to air pollution and the incidence of Kawasaki disease

**DOI:** 10.1038/s41598-022-07081-y

**Published:** 2022-03-01

**Authors:** Ni-Chun Kuo, Chien-Heng Lin, Ming-Chih Lin

**Affiliations:** 1 Children’s Medical Center, Taichung Veterans General Hospital, 1650 Taiwan Boulevard Sec. 4, Taichung, 40705 Taiwan, ROC; 2 Department of Medical Research, Taichung Veterans General Hospital, Taichung, Taiwan, ROC; 3 Department of Post‐Baccalaureate Medicine, College of Medicine, National Chung Hsing University, Taichung, Taiwan, ROC; 4 School of Medicine, National Yang-Ming University, Taipei, Taiwan, ROC; 5 Department of Food and Nutrition, Providence University, Taichung, Taiwan, ROC; 6 School of Medicine, Chung Shan Medical University, Taichung, Taiwan, ROC

**Keywords:** Environmental impact, Risk factors, Epidemiology, Paediatric research

## Abstract

Kawasaki disease (KD) is the most common form of acquired pediatric cardiac disease in the developed world. However, its etiology is still unclear. Epidemiological studies have shown that air pollution is a plausible risk factor in stimulating oxidative stress, inducing inflammation and causing autoimmune diseases. This study aims to assess the connections between prenatal and early life air pollution exposure to the incidence of KD. The main data source of this nationwide longitudinal study was the National Health Insurance Research Database (NHIRD) of Taiwan. NHIRD was linked with Taiwan Maternal and Child Health Database to establish the link between mothers and children. In total, 4192 KD cases involving children under 6 years of age were identified between January 2004 and December 2010. Children in the control group were randomly selected at a 1:4 ratio and matched using their age and index year. Integrated data for the air pollutants were obtained from 71 Environmental Protection Agency monitoring stations across Taiwan. Patients who had main admission diagnosis of KD and subsequently received intravenous immunoglobulin treatment were defined as incidence cases. Ambient exposure, including pollutant standards index (PSI), carbon monoxide (CO), nitric oxide (NO), nitric dioxide (NO_2_), and nitrogen oxide (NOx) during pregnancy were all positively associated with KD incidence. Conversely, ozone (O_3_) exposure had a negative correlation. Exposure to CO, NO, NO_2,_ and NOx after childbirth remained consistent with regards to having a positive association with KD incidence. Exposure to PSI and O_3_ after delivery displayed no significant association with KD. Both prenatal and postnatal cumulative CO, NO, NO_2_, and NOx exposure had a dose dependent effect towards increasing KD incidence. Certain prenatal and early life air pollutant exposure may increase the incidence of KD.

## Introduction

Kawasaki disease (KD), an acute form of multi-systemic vasculitis, predominantly occurs amongst preschool children. KD is characterized by prolonged fever and systemic principal features including conjunctivitis, cervical lymphadenopathy, changes in extremities, polymorphous skin rashes, and oral mucosa changes. KD is also the most common form of acquired pediatric cardiac disease in the developed world due to the sequelae of coronary artery lesions if not treated promptly^1,2^. The etiology of KD remains unclear. After decades of research and epidemiological observations, an environmental trigger causing an inflammatory process in genetically predisposed individuals is the most reasonable mechanism of pathogenesis^3^.

Epidemiological studies have shown air pollution to be a plausible risk factor in stimulating oxidative stress, inducing inflammation and causing autoimmune diseases^4,5^. Air pollution has also been reported to be associated with Kawasaki disease^6^. Geographically evaluating exposure to air pollution parameters such as ozone (O_3_), nitrogen oxides (NO_x_), sulfur dioxide (SO_2_), carbon monoxide (CO), particulate matter < 2.5 μm in diameter (PM2.5), and particulate matter < 10 μm in diameter (PM10) may influence the susceptibility of children with Kawasaki disease^7,8^. However, studies regarding the association between Kawasaki disease and air pollution are sparse and their results are divergent^6,9,10^. Furthermore, the effect of prenatal air pollution exposure on the incidence of KD has never been reported. This nationwide longitudinal study aims to assess the correlation between prenatal and early-life air pollution exposure to the development of Kawasaki disease.

## Methods

### Data source

A single-payer National Health Insurance (NHI) program with mandatory enrollment was launched in Taiwan in 1995, with a coverage rate of 99.99% of Taiwan’s population. The National Health Insurance Research Database (NHIRD) was established based upon the claim data of NHI in 2002^11,12^. Since 2015, the NHIRD has been further integrated with other health-related databases located at the Health and Welfare Data Center (HWDC) of Taiwan’s Ministry of Health and Welfare (MOHW)^11,13^. The main source of data for analysis is found in the inpatient expenditures by admission (DD) files from the NHIRD. The NHIRD was further linked with the Taiwan Maternal and Child Health Database (MCHD) maintained by Taiwan’s Health Promotion Administration (HPA) to help establish the link between mothers and children. Investigators are required to conduct on-site analysis for epidemiological studies, as Taiwan’s Ministry of Health and Welfare (MOHW) endeavors to protect privacy and validate its database reliability. All diagnoses in the NHIRD are coded through use of the International Classification of Diseases, Ninth Revision, Clinical Modification (ICD-9-CM) format.

### Study design

**Figure 1:**
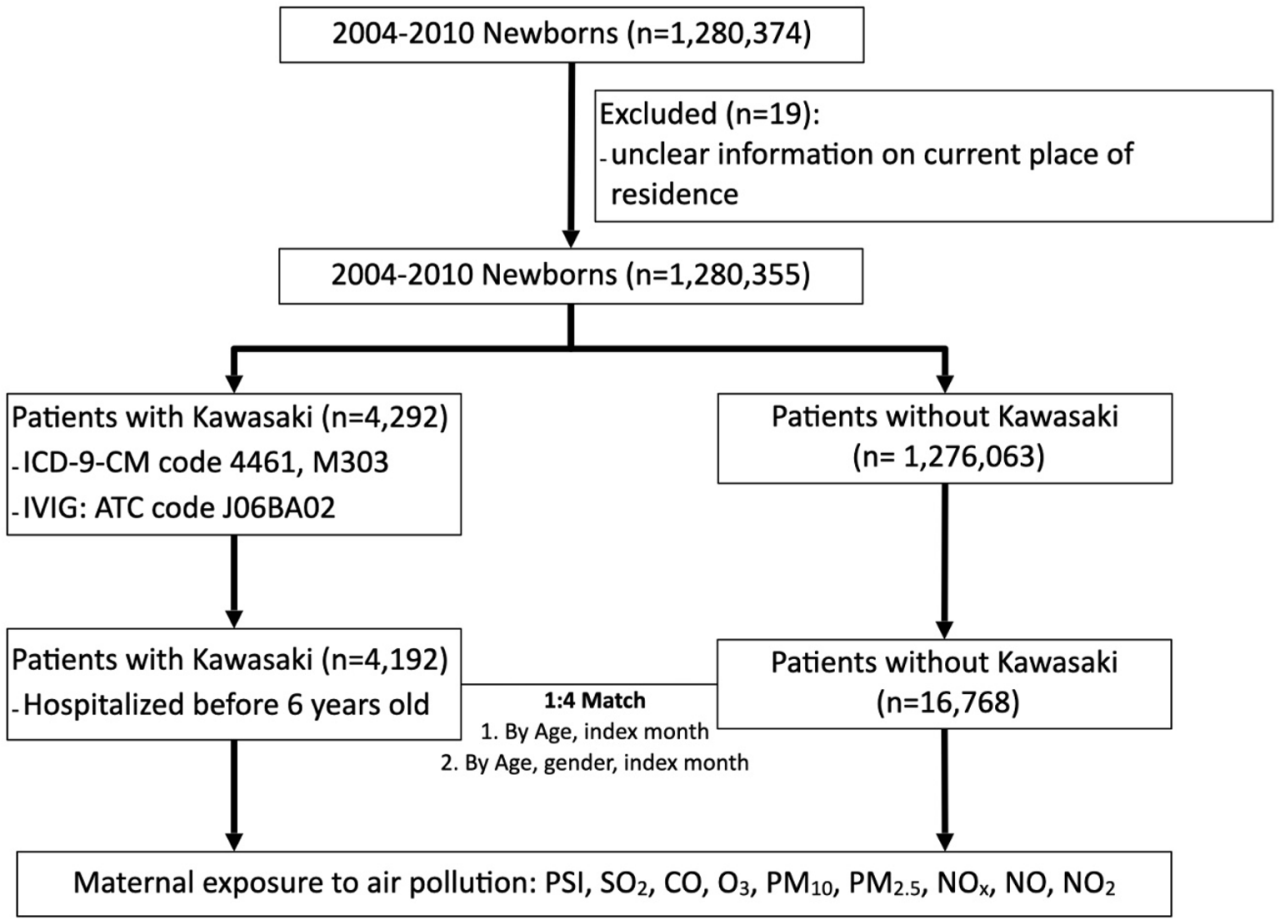
Composition of the study cohort.

Figure 1 presents the flowchart of the patient enrollment. Our study identified a cohort of 4192 Kawasaki patients who were hospitalized at an age under 6 years between January 2004 to December 2010. Patients were regarded as incidence cases of KD when their main admission diagnosis was KD (ICD-9-CM 446.1), and subsequently received treatment with intravenous immunoglobulin (IVIG, ATC code: J06BA02)^14^. Control groups matched through both age and index month were randomly chosen at a case-to-control ratio of 1:4. To test the robustness of our analysis, we chose another control group matched by age, index month, and gender. Patients’ gender, date of diagnosis, age, maternal information and ambient air pollution where they were living were all retrieved. All subjects were followed from the index date until either the diagnosis of KD or the end of the 6-year follow-up. This study protocol was approved, and the need of informed consent was waived by the institutional review board of Taichung Veterans General Hospital (CE17178A-3). All methods were performed in accordance with the relevant guidelines and regulations of Scientific Reports.

### Assessment of exposure to air pollutants

Taiwan Environmental Protection Agency (TEPA) established the Taiwan Air Quality Monitoring Network in 1994. This network continuously monitored the air quality of Taiwan with a total of 76 stations and reported the concentrations of SO_2_, NO_2_, O_3_, CO, and PM10 hourly. The selection of the site of air quality monitoring stations is established after careful planning and design based on emission of pollutants, meteorological status and distribution of air quality concentration in each area, etc. According to different monitoring purposes, monitoring stations are divided into the following 6 types: (1) general stations, containing 60 sites, were installed in populous sites that can represent the distribution of air quality of a larger area and reflect the air quality status of people’s daily life; (2) traffic air quality monitoring stations, containing 6 sites, were installed at areas with heavy traffic so to reflect the air quality to which the pedestrians are exposed; (3) industrial air quality monitoring stations, containing 5 sites, were installed in leeward side of the prevailing wind in industrial areas, so to understand the influence of industrial pollution; (4) National park monitoring stations, were installed at appropriate sites in reserved areas; (5) background air quality monitoring stations, were installed in areas with less pollution or in the windward side of the prevailing wind in the total quantity control zones. Taiwan is divided into 22 primary administrative divisions and is further divided into 368 townships. In our study, National Park monitoring stations and background air quality monitoring stations were excluded due to low density dwellings. The remaining 71 stations provided the exposure level of air pollutants in our study population. Residential postal codes of our study population scattered in 368 subdivisions. The data of each air pollutant exposure were determined by the residential postal codes of 368 townships in accordance with one air quality monitoring station. Taiwan Air Quality Monitoring Network publishes air quality report annually and provide real-time air quality indicator on the website^15^. In our study, the calculation results represented for each pollutants exposure were monthly average concentrations corresponding to 1-h SO2, 8-h CO, 8-h CO2, 8-h O3, 24-h PM10, 24-h PM2.5, 1-h NOx, 1-h NO and 1-h NO2 monitoring continuously. PSI, which was calculated according to five sub-indexes (SO2, NO2, O3, CO, and PM10), was presented as a scale extending from 0 (healthy) to 500 (extremely unhealthy). The air pollutant measurements, which focus on the consistency in air quality assessment, are derived from Taiwan EPA monitoring stations. As an indicator of average prenatal exposure, we used monthly average concentrations of air pollutants assigned to mother’s exposure for 10 consecutive months (40 weeks) before birth and the mother’s residential postal code at time of delivery. As an indicator of average early childhood, we used monthly average concentrations of air pollutants derived from the child’s exposure until KD was diagnosed (index date) and the child’s residential postal code at time of admission to hospital.

### Statistics

We performed all analyses using the SAS statistical package (version 9.3; SAS Institute, Cary, North Carolina, USA). All quantitative data were expressed as either frequency and percentage or mean and standard deviation. Continuous variables were compared using the Student’s t-test, while Pearson’s chi-square test was applied for categorical data. A multiple logistic regression model was applied for adjusting the potential confounding factors between the correlation of air pollution and KD incidence. The risks for both KD and air pollution exposure amount per interquartile range were calculated to determine the dose dependent effect. To test the robustness of our analysis, we further used conditional logistic regression models to analyze the second group of controls who were matched by index month, age, and gender.

### Ethics approval and consent to participate

This study protocol was approved by the institutional review board of Taichung Veterans General Hospital (CE17178A-3). Consent to participate in this study was waived by the review board.

## Results

### Baseline characteristics of KD and age-matched controls

A total of 4192 KD admissions below the age of 6 years were identified from our study population between January 2004 to December 2010. A total of 16,768 children (1:4) without KD, matched by age and index date were randomly selected as the control group. KD occurred mostly at the age of 0–1 year, with a significant male predominance (62%). The KD group tended to have more mothers at an advanced age, underwent a vaginal delivery, and experienced maternal allergic rhinitis as well as maternal atopic dermatitis. There were no clear disproportionate differences between birth weight and preterm delivery (Table 1). The demographic data of the second control group was summarized in the Supplement File. Regarding air pollution exposure during pregnancy, levels of exposure to PSI, CO, O_3_, and NOx were higher in patients with KD than in the matched controls (p < 0.05). Interestingly, levels of exposure to PSI and O_3_ were inconsistent with the previously mentioned results regarding air pollution exposure after pregnancy (Table 2).

**Table 1:** Baseline characteristics of Kawasaki disease and control groups.

Characteristic	Non-KD group (n = 16,768)	KD group (n = 4192)	*p*-value
	n (%)	n (%)	
**Neonatal age**			1.000
0–1	6868 (41)	1717 (41)	
1–2	5028 (30)	1257 (30)	
2–3	2332 (13.9)	583 (13.9)	
3–4	1180 (7)	295 (7)	
4–5	824 (4.9)	206 (4.9)	
5–6	536 (3.2)	134 (3.2)	
**Neonatal gender**			< 0.001
Female	8029 (47.9)	1593 (38)	
Male	8739 (52.1)	2599 (62)	
**Birth weight (g)**			0.78
≥ 2500	15,680 (93.5)	3925 (93.6)	
< 2500	1088 (6.5)	267 (6.4)	
**Maternal age**			0.003
< 35	14,564 (86.9)	3567 (85.1)	
≥ 35	2204 (13.1)	625 (14.9)	
**Mode of delivery**			0.003
Vaginal delivery	10,940 (65.2)	2631 (62.8)	
Cesarean section	5828 (34.8)	1561 (37.2)	
**Preterm delivery**			0.46
≥ 37 weeks	15,462 (92.2)	3851 (91.9)	
< 37 weeks	1306 (7.8)	341 (8.1)	
**Maternal comorbidity**			
Asthma	301 (1.8)	84 (2)	0.37
Allergic rhinitis	1614 (9.6)	458 (10.9)	0.012
Atopic dermatitis	578 (3.4)	195 (4.7)	< 0.001

* KD* Kawasaki Disease; *Non-KD* non-Kawasaki Disease .

**Table 2:** Level of exposure to air pollutants during pregnancy and in early life.

	Non KD group (n = 16,768)	KD group (n = 4192)	*p* value
**Level of exposure to air pollutants during pregnancy, mean (SD)**			
PSI	57.16 (7.44)	57.41 (7.38)	0.05
SO_2_ (ppb)	4.43 (1.49)	4.45 (1.49)	0.40
CO (ppm)	0.51 (0.11)	0.52 (0.11)	< 0.001
CO_2_ (ppm)	917.13 (815.62)	887.54 (578.42)	0.12
O_3_ (ppb)	28.71 (3.04)	28.59 (3)	0.018
PM10 (μg/m^3^)	59.18 (13.57)	59.37 (13.37)	0.40
PM2.5 (μg/m^3^)	52.26 (66.82)	51.6 (61.98)	0.56
NOx (ppb)	24.67 (6.76)	25.17 (7.01)	< 0.001
NO (ppb)	6.09 (2.52)	6.28 (2.62)	< 0.001
NO_2_ (ppb)	18.59 (4.5)	18.89 (4.65)	< 0.001
**Level of exposure to air pollutants in early life, mean (SD)**			
PSI	56.49 (8.16)	56.75 (7.60)	0.05
SO_2_ (ppb)	4.39 (1.50)	4.40 (1.45)	0.70
CO (ppm)	0.49 (0.10)	0.50 (0.10)	< 0.001
CO_2_ (ppm)	915.6 (1377.3)	924.7 (1474.2)	0.83
O_3_ (ppb)	28.79 (3.80)	28.72 (2.93)	0.25
PM10 (μg/m^3^)	58.71 (15.52)	58.90(14.84)	0.48
PM2.5 (μg/m^3^)	43.47 (77.50)	44.91 (95.42)	0.37
NOx (ppb)	23.95 (7.36)	24.47 (8.25)	< 0.001
NO (ppb)	5.89 (2.64)	6.06 (2.65)	< 0.001
NO_2_ (ppb)	18.06 (4.95)	18.41 (5.89)	< 0.001

*CO* carbon monoxide, *CO*_*2*_ carbon dioxide, *KD groups* Kawasaki disease, *Non-KD* non-Kawasaki disease, *NO* nitric oxide, *NO*_*2*_ nitric dioxide, *NO*_*x*_ nitrogen oxide, *O*_*3*_ ozone, *PM2.5* particulate matter 2.5 uM, *PM10* particulate matter 10 uM, *PSI* pollutant standards index, *SO*_*2*_ sulfur dioxide.

### Multiple logistic regression analysis for air pollutant and risk of KD

Table 3 summarizes the results of multiple logistic regression models of PSI, CO, O_3_, NOx, NO and NO_2_ exposure both during pregnancy and after delivery. After adjustment for possible confounders, ambient exposure to PSI, CO and NOx was positively associated with KD during pregnancy; however, O_3_ had a negative correlation. Additionally, ambient exposure to CO, NOx, NO and NO_2_ after delivery remained a consistent positive association with KD. In contrast, exposure to PSI and O_3_ after delivery revealed no significant association with KD occurrence.

**Table 3:** Multiple logistic regression analysis for air pollutant and risk of Kawasaki disease.

Characteristic	Air pollution exposure during pregnancy				Air pollution exposure after delivery			
	OR	95% CI		p-value	OR	95% CI		p-value
PSI	1.01	1.00	1.01	0.05	1.00	1.00	1.01	0.12
CO (ppm)	1.81	1.34	2.46	< 0.001	1.64	1.18	2.27	0.003
O_3_ (ppb)	0.99	0.98	1.00	0.019	1.00	0.99	1.01	0.34
NOx (ppb)	1.01	1.01	1.02	< 0.001	1.01	1.00	1.01	0.001
NO (ppb)	1.03	1.02	1.04	< 0.001	1.02	1.01	1.03	0.003
NO_2_ (ppb)	1.01	1.01	1.02	< 0.001	1.01	1.00	1.02	0.001

Model adjusted for age, gender, birth weight, mother age, mode of delivery, preterm delivery and maternal comorbidity.

*PSI* pollutant standards index, *CO* carbon monoxide, *O3* ozone, *NO*_*x*_ nitrogen oxide, *NO* nitric oxide, *NO*_*2*_ nitric dioxide, *OR* odds ratio, *CI* confidence interval.

### Conditional logistic regression analysis of factors associated with KD admissions

To test the robustness of our analysis, we further selected another control group by matching age, index month, and gender. Alternatively, we adapted a conditional logistic regression model for confounders adjustment. Table 4 summarizes the results of conditional logistic regression models of PSI, CO, O_3_, NOx, NO and NO_2_ exposure both during pregnancy and after delivery. After adjustment for possible confounders, exposure measured by PSI changed from borderline significance to non-significance. Ambient exposure to CO and NOx was still positively associated with KD during pregnancy. However, O_3_ had a non-significant correlation. Interestingly, PSI after delivery became significant when analyzed by conditional logistic regression models. Ambient exposure to CO, NOx, NO and NO_2_ after delivery remained a consistent positive association with KD.

**Table 4:** Conditional logistic regression analysis for air pollutant and risk of Kawasaki disease.

Characteristic	Air pollution exposure during pregnancy				Air pollution exposure after delivery			
	OR	95% CI		p-value	OR	95% CI		p-value
PSI	1.00	1.00	1.01	0.07	1.01	1.00	1.01	0.02
CO (ppm)	1.67	1.23	2.28	0.001	1.61	1.16	2.22	0.004
O_3_ (ppb)	0.99	0.98	1.01	0.26	1.00	0.99	1.01	0.61
NOx (ppb)	1.01	1.00	1.01	0.002	1.01	1.00	1.01	0.001
NO (ppb)	1.02	1.01	1.03	0.002	1.02	1.01	1.03	0.001
NO_2_ (ppb)	1.01	1.00	1.02	0.004	1.01	1.00	1.02	0.002

Model adjusted for birth weight, mother age, mode of delivery, preterm delivery and maternal comorbidity.

*PSI* pollutant standards index, *CO* carbon monoxide, *O3* ozone, *NO*_*x*_ nitrogen oxide, *NO* nitric oxide, *NO*_*2*_ nitric dioxide, *OR* odds ratio, *CI* confidence interval.

### Dose dependent effect of air pollution on risk of KD

**Figure 2:**
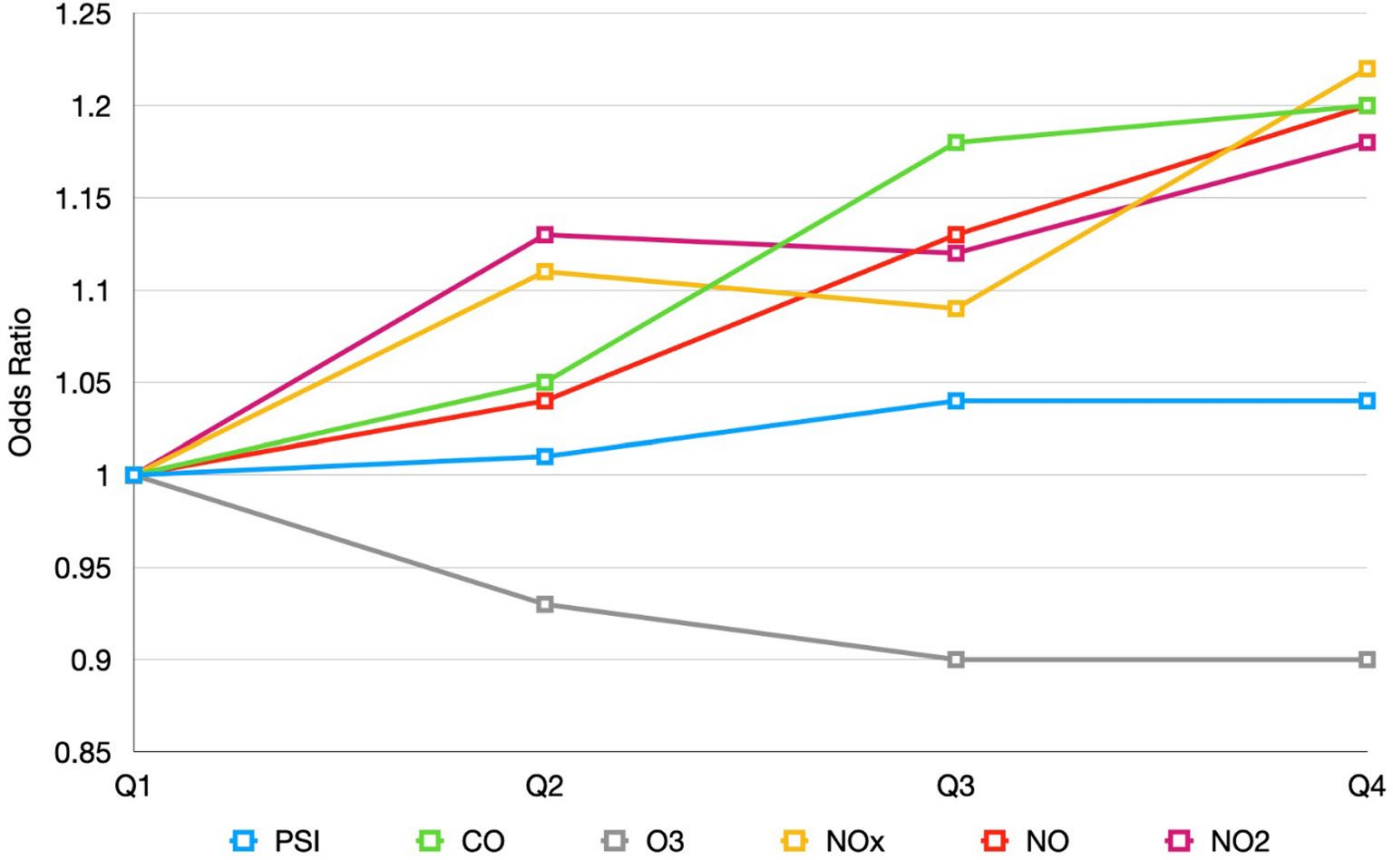
Quartiles of prenatal cumulative air pollutant exposure to the risk of Kawasaki disease.

**Figure 3:**
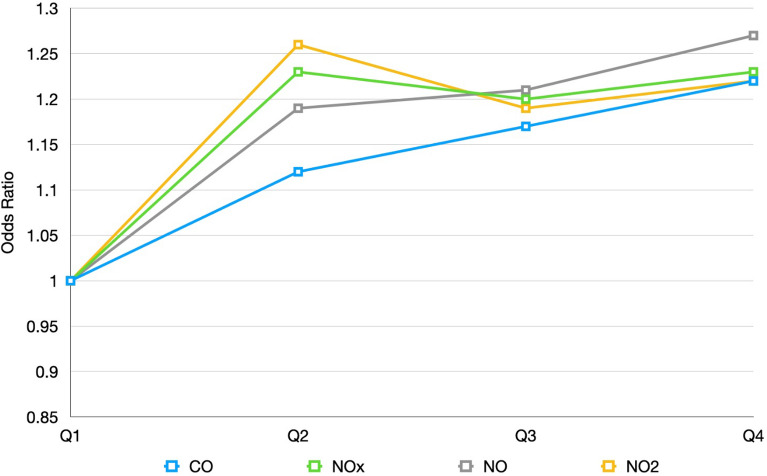
Quartiles of early life cumulative air pollutant exposure to the risk of Kawasaki disease.

The dose dependent effect of air pollution was further analyzed according to the quartiles of the cumulative amounts of air pollutants. For exposure during pregnancy, odds ratios of KD became larger as increase in cumulative amounts of pollutants. Liner trends can be observed for CO, NO, NO_2_, and NOx (Fig. 2). For early life exposure, similar trends could also be observed for CO, NO, NO_2_, and NOx (Fig. 3). On the contrary, both prenatal and postnatal cumulative O_3_ exposure had a negative linear trend towards decreasing KD incidence (Figs. 2, 3).

## Discussion

This population-based nationwide longitudinal cohort study provides preliminary evidence that exposure to certain types of air pollution, both prenatally and during early-life, may contribute to the development of KD in children.

KD has long been considered an immune disorder. The strong association between KD and allergic diseases can be demonstrated by elevated serum IgE levels in KD patients^16^. Activation of Th1 immune reactions such as interferon-Gamma, tumor necrosis factor-alpha, IL-1 and IL-10, as well as Th2-mediated immune reactions, such as IL-4, IL-5, and IL-13 have also been proven during the acute phase of KD^17,18^. Furthermore, immune complexes are now considered to be playing an important role in the pathogenesis of KD^19^. Attention has been given to the possibility of ambient air pollutions triggering KD^20,21^. Both NOx and CO are predominantly traffic-related emissions^22,23^. NOx includes odorless NO and pungently odorous NO_2_. NOx is a known traffic pollutant which affects the nervous system, thus diminishing forced expiratory flow, and increasing respiratory symptoms in children^23,24^. Human hemoglobin (Hb) has 218 times the affinity to CO than oxygen. Once carboxyhemoglobin (COHb) is formed, it reduces oxygen capacity in blood (hypoxemia), possibly causing low oxygen in tissues (hypoxia)^25^. CO-associated ambient air pollution is shown to cause a hypoxia status resulting in cell damage in the heart, lung, muscle, and brain, along with the immune and nervous systems^26,27^. Prenatal and/or postnatal exposures to air pollution may induce immune dysregulation, systemic inflammation and oxidative stress^28^. Those effects hold biological plausibility for the development of KD. Exposure to traffic-related pollutants could cause higher allergic susceptibility for children early in life^29^. Increased allergic disease susceptibility due to traffic-related air pollutants may also contribute to the risk of KD^29–31^.

Another interesting finding of this study is that O_3_ acts as a protective factor for KD in those females who are exposed during pregnancy. Ozone pollution is mainly caused by traffic-related emissions and their interaction with sunlight. Exposure to ozone results in a secondary increase in reactive oxygen species, causing an oxidative stress state in the organism which triggers an inflammatory state^32^. Hazards to epithelial injury in the tracheobronchial tree have been identified in those who have experienced short-term exposure, as well as exposure levels as low as 0.15 ppm ozone in monkeys^33^. As described in the time-stratified case-crossover study in Taiwan, exposure to ground-level ozone may increase the risk of KD during childhood^10^. Maternal exposures to ozone concentrations may attribute to allergic immune responses in their offspring^34^. However, maternal exposure to ozone was negatively associated with the incidence of KD in this study. The possible explanation for this is that the effect of O_3_ exposure is dose-dependent: high dosages stimulate severe oxidative stress resulting in inflammatory response and tissue injury, whereas low O_3_ concentrations induce a moderate oxidative stress activating protective antioxidant pathways^8^. The reason for maternal exposure to O_3_ during pregnancy reducing KD may be a result of the threshold effect, in that the hazardous effects of O_3_ may not be obvious below its harmful cut-off value. Instead, low O_3_ concentrations trigger maternal anti-oxidative system, leading to the protective role of the antioxidant. Further clinical and experimental studies on this relationship are still needed in order to identify any direct role O_3_ may play.

Previous reports have suggested that exposure to Particulate Matter (PM) air pollution contributes to cardiovascular and pulmonary morbidity, as well as mortality, by stimulating the onset of the systemic inflammatory response^35,36^. However, available literature has shown there are no significant associations between fine and coarse particulate matter exposure and KD occurrence^6,9,10,37^. SO_2_, PM2.5 and PM10 are mainly derived from stationary fossil combustion processes. In this study, fossil fuel combustion-related air pollution brought upon no significant effects on the incidence of KD.

Male gender is a well-known risk factor for KD^38^. Because misclassification of gender in the database is very rare and it is easy to control in the statistical model, we did not apply “sex” as a matching factor. Moreover, we would like to observe whether the gender factor took a role in the relationship of air pollution exposure and KD incidence. Other potential confounders may also be considered for the matching process to control the confounding effects. As the same reason, we would like to control it in the statistical model rather than matching them. In the contrary, because KD incidence waxes and wanes as seasons change, it is hard to compare if we did not choose children in the control group by the same birth month^14^. Furthermore, those who developed KD earlier tend to have a higher exposure level (air pollution concentrations are likely to decrease along time) and a greater variation. These are reasons why we match case and control groups by index month. Although air pollution level presenting by absolute values cannot tell significant differences clearly by the number itself and standardized values convey obvious concrete concepts, continuous variable values provide more information than the categorical values that classified air pollutants level into scales. Moreover, continuous variables own more statistical power to detect differences among groups. Previous literature also presented the pollution level as original values^39^. Only the data of PSI were presented as a scale extending from 0 (healthy) to 500 (extremely unhealthy) since PSI is calculated according to five sub-index (SO2, NO2, O3, CO, and PM10). Previous articles regarding prenatal and early life exposure to air pollution and KD demonstrated the confounding factors such as age, gender, birth weight, mother age, mode of delivery and preterm delivery, which were then applied in our study^21,28^. Maternal comorbidity including asthma, allergic rhinitis and atopic dermatitis were selected in the list of confounders since previous literature illustrated that children born from mothers with asthma and allergic rhinitis had a higher risk of developing KD^40^. In our study, we control those potential confounders in multiple logistic regression models to see the independent effect of air pollution itself.

Our study has certain limitations. Firstly, the average exposure level of air pollutants was calculated according to residential postal codes. The residential postal codes typically matched along with one block-face in urban areas but was larger in rural areas due to low population density. The study cannot determine the actual distance between the residential postal codes and air quality monitoring stations nor distinguished difference in coverage from urban to rural areas. The air quality monitoring station that assigned to each postal code was based on administrative districts, which may not be the closest to each participant. Also, migration from one city to another could lead to misclassifications in levels of exposure. Secondly, seasonal weather patterns such as temperature, humidity, and wind were not considered in our study. Seasonal weather patterns may influence the composition of environmental pollutants due to fluctuating stratified atmospheric structures^41^. KD incidence in Taiwan also follows a significant seasonal trend^10,14^. Under the influence of the Asian continental anticyclone system, a strong northeasterly monsoonal flow causes more favorable conditions for disease onset, which could possibly be associated with transboundary air pollution and agents^42^. Thirdly, urbanization might be associated with easier access to healthcare, and therefore possessed a higher positive diagnosis rate. The National Health Insurance, characterized by good accessibility and comprehensive population coverage, was launched in Taiwan since 1995. In addition, Taiwan is a small island. As a result, urban–rural gap regarding medical accessibility is relatively narrow. Although higher positive diagnosis rate may be existed in urban area, it should not be a major concern. And finally, the present study has considered only relatively important air pollutants. However, some other airborne agents such as spores and microorganisms have also been reported to be associated with the occurrence of KD^42^. This study used outdoor concentration with fixed ambient monitoring system to analyze the cumulative exposure. However, the real exposure can be in indoors because most people have spent their time in indoors. Temperature and humidity should also be considered as potential confounders in multiple logistic regression models. However, the data were not recorded in those monitoring stations. Furthermore, the biological mechanism of how prenatal air pollution affects the incidence of Kawasaki disease is still unclear.

## Conclusions

Certain types of prenatal and early life air pollutant exposure may increase the incidence of KD. Further prospective studies and additional data taken from countries other than Taiwan are still needed in order to elucidate on this causal relationship.

## Supplementary Information

Supplementary Information.

## Data Availability

The datasets presented in this article are not readily available because data release is not allowed by the National Health Insurance Research Database. Requests to access the datasets should be directed to Dr. Chien-Heng Lin/epid@ms39.hinet.net.

## References

[1] Liang CD, Kuo HC, Yang KD, Wang CL, Ko SF (2009). Coronary artery fistula associated with Kawasaki disease. Am. Heart J..

[2] de Graeff N (2019). European consensus-based recommendations for the diagnosis and treatment of Kawasaki disease—The SHARE initiative. Rheumatology (Oxford).

[3] Burgner D, Harnden A (2005). Kawasaki disease: What is the epidemiology telling us about the etiology?. Int. J. Infect. Dis..

[4] Sun G (2016). Association between air pollution and the development of rheumatic disease: A systematic review. Int. J. Rheumatol..

[5] Wu W, Jin Y, Carlsten C (2018). Inflammatory health effects of indoor and outdoor particulate matter. J. Allergy Clin. Immunol..

[6] Zeft AS (2016). Kawasaki disease and exposure to fine particulate air pollution. J. Pediatr..

[7] Corinaldesi E (2020). Environmental factors and Kawasaki disease onset in Emilia-Romagna, Italy. Int. J. Environ. Res. Public Health..

[8] Lodovici M, Bigagli E (2011). Oxidative stress and air pollution exposure. J. Toxicol..

[9] Lin Z (2017). Ambient air pollution, temperature and Kawasaki disease in Shanghai, China. Chemosphere.

[10] Jung C-R, Chen W-T, Lin Y-T, Hwang B-F (2017). Ambient air pollutant exposures and hospitalization for Kawasaki disease in Taiwan: A case-crossover study (2000–2010). Environ. Health Perspect..

[11] Hsieh CY (2019). Taiwan's National Health Insurance Research Database: Past and future. Clin. Epidemiol..

[12] Lin MC, Lai MS (2009). Pediatricians' role in caring for preschool children in Taiwan under the national health insurance program. J. Formos Med. Assoc..

[13] Lin LY, Warren-Gash C, Smeeth L, Chen PC (2018). Data resource profile: The National Health Insurance Research Database (NHIRD). Epidemiol. Health.

[14] Lin MC, Lai MS, Jan SL, Fu YC (2015). Epidemiologic features of Kawasaki disease in acute stages in Taiwan, 1997–2010: Effect of different case definitions in claims data analysis. J. Chin. Med. Assoc..

[15] *Taiwan Air Quality Monitoring Network.* Environmental Protection Administration Executive Yuan, R.O.C. (Taiwan). https://airtw.epa.gov.tw/ENG/default.aspx. Accessed 22 September 2021.

[16] Abe J (2008). Elevated granulocyte colony-stimulating factor levels predict treatment failure in patients with Kawasaki disease. J. Allergy Clin. Immunol..

[17] Kuo HC, Yang KD, Chang WC, Ger LP, Hsieh KS (2012). Kawasaki disease: An update on diagnosis and treatment. Pediatr. Neonatol..

[18] Lin IC (2012). Augmented TLR2 expression on monocytes in both human Kawasaki disease and a mouse model of coronary arteritis. PLoS ONE.

[19] Menikou S, Langford PR, Levin M (2019). Kawasaki disease: The role of immune complexes revisited. Front. Immunol..

[20] Rodó X (2014). Tropospheric winds from northeastern China carry the etiologic agent of Kawasaki disease from its source to Japan. Proc. Natl. Acad. Sci. U.S.A..

[21] Buteau S (2020). Association between Kawasaki disease and prenatal exposure to ambient and industrial air pollution: A population-based cohort study. Environ. Health Perspect..

[22] Lee YL (2003). Climate, traffic-related air pollutants and allergic rhinitis prevalence in middle-school children in Taiwan. Eur. Respir. J..

[23] Manisalidis I, Stavropoulou E, Stavropoulos A, Bezirtzoglou E (2020). Environmental and health impacts of air pollution: A review. Front. Public Health.

[24] Wjst M (1993). Road traffic and adverse effects on respiratory health in children. BMJ (Clin. Res.).

[25] Rodkey FL, O’neal JD, Collison HA (1969). Oxygen and carbon monoxide equilibria of human adult hemoglobin at atmospheric and elevated pressure. Blood.

[26] Lv Y-W (2013). Understanding the pathogenesis of Kawasaki disease by network and pathway analysis. Comput. Math. Methods Med..

[27] Levy RJ (2015). Carbon monoxide pollution and neurodevelopment: A public health concern. Neurotoxicol. Teratol..

[28] Yorifuji T, Tsukahara H, Kashima S, Doi H (2018). Intrauterine and early postnatal exposure to particulate air pollution and Kawasaki disease: A Nationwide Longitudinal Survey in Japan. J. Pediatr..

[29] Tsai YJ (2013). The association between Kawasaki disease and allergic diseases, from infancy to school age. Allergy Asthma Proc..

[30] Wei CC (2014). Increased risk of Kawasaki disease in children with common allergic diseases. Ann. Epidemiol..

[31] Huang PY, Huang YH, Guo MM, Chang LS, Kuo HC (2020). Kawasaki disease and allergic diseases. Front. Pediatr..

[32] Rivas-Arancibia S (2010). Oxidative stress caused by ozone exposure induces loss of brain repair in the hippocampus of adult rats. Toxicol. Sci..

[33] Hyde DM (1992). Ozone-induced acute tracheobronchial epithelial injury: Relationship to granulocyte emigration in the lung. Am. J. Respir. Cell Mol. Biol..

[34] Sharkhuu T, Doerfler DL, Copeland C, Luebke RW, Gilmour MI (2011). Effect of maternal exposure to ozone on reproductive outcome and immune, inflammatory, and allergic responses in the offspring. J. Immunotoxicol..

[35] Brook RD (2010). Particulate matter air pollution and cardiovascular disease. Circulation.

[36] Chen R (2015). Size-fractionated particulate air pollution and circulating biomarkers of inflammation, coagulation, and vasoconstriction in a panel of young adults. Epidemiology.

[37] Corinaldesi E (2020). Environmental factors and Kawasaki disease onset in Emilia-Romagna, Italy. Int. J. Environ. Res. Public Health.

[38] McCrindle BW (2017). Diagnosis, treatment, and long-term management of Kawasaki disease: A scientific statement for health professionals from the American Heart Association. Circulation.

[39] Tang KT (2017). Adult atopic dermatitis and exposure to air pollutants—A nationwide population-based study. Ann. Allergy Asthma Immunol..

[40] Chu HW, Lin CH, Lin MC, Hsu YC (2021). Increased risk of Kawasaki disease in infants born of mothers with immune disorders. Front. Pediatr..

[41] Huang W-R, Wang S-Y (2014). Impact of land–sea breezes at different scales on the diurnal rainfall in Taiwan. Clim. Dyn..

[42] Cheng F-Y, Hsu C-H (2019). Long-term variations in PM2.5 concentrations under changing meteorological conditions in Taiwan. Sci. Rep..

