# Supplementary material for: Prenatal and early life exposure to air pollution and the incidence of Kawasaki disease
Source: Sci Rep. 2022 Mar 1;12:3415. doi: 10.1038/s41598-022-07081-y (PMC8888747; doi:10.1038/s41598-022-07081-y)
Supplement: Supplementary file 1 — Supplementary Information. [file 41598_2022_7081_MOESM1_ESM.docx]

Baseline Characteristics of Kawasaki disease and control groups (matched by age, index month and gender)

| **Characteristic** | **Non KD group** | **KD group** | **Total** | ***P*-value** |
| --- | --- | --- | --- | --- |
|  | **(n=16768)** | **(n=4192)** |  |  |
|  | **n (%)** | **n (%)** |  |  |
| **Neonatal age** |  |  |  | 1.000 |
| 0-1 | 6868 (41) | 1717 (41) | 8585 |  |
| 1-2 | 5028 (30) | 1257 (30) | 6285 |  |
| 2-3 | 2332 (13.9) | 583 (13.9) | 2915 |  |
| 3-4 | 1180 (7) | 295 (7) | 1475 |  |
| 4-5 | 824 (4.9) | 206 (4.9) | 1030 |  |
| 5-6 | 536 (3.2) | 134 (3.2) | 670 |  |
| **Neonatal gender** |  |  |  | 1.000 |
| Female | 6372 (38) | 1593 (38) | 7965 |  |
| Male | 10396 (62) | 2599 (62) | 12995 |  |
| **Birth weight (g)** |  |  |  | 0.944 |
| ≥2500 | 15695 (93.6) | 3925 (93.6) | 19620 |  |
| <2500 | 1073 (6.4) | 267 (6.4) | 1340 |  |
| **Maternal age** |  |  |  | 0.002 |
| <35 | 14580 (87) | 3567 (85.1) | 18147 |  |
| ≥35 | 2188 (13) | 625 (14.9) | 2813 |  |
| **Mode of delivery** |  |  |  | <0.001 |
| Vaginal delivery | 11022 (65.7) | 2631 (62.8) | 13653 |  |
| Cesarean section | 5746 (34.3) | 1561 (37.2) | 7307 |  |
| **Preterm delivery** |  |  |  | 0.970 |
| ≥37 weeks | 15407 (91.9) | 3851 (91.9) | 19258 |  |
| <37 weeks | 1361 (8.1) | 341 (8.1) | 1702 |  |
| **Maternal comorbidity** |  |  |  |  |
| Asthma (AS) | 351 (2.1) | 84 (2) | 435 | 0.716 |
| Allergic rhinitis (AR) | 1731 (10.3) | 458 (10.9) | 2189 | 0.254 |
| Atopic dermatitis (AD) | 559 (3.3) | 195 (4.7) | 754 | <0.001 |
